# PEG-OligoRNA Hybridization of mRNA for Developing Sterically Stable Lipid Nanoparticles toward In Vivo Administration

**DOI:** 10.3390/molecules24071303

**Published:** 2019-04-03

**Authors:** Shota Kurimoto, Naoto Yoshinaga, Kazunori Igarashi, Yu Matsumoto, Horacio Cabral, Satoshi Uchida

**Affiliations:** 1 Department of Bioengineering, Graduate School of Engineering, The University of Tokyo, 7-3-1 Hongo, Bunkyo-ku, Tokyo 113-8655, Japan; kurimoto@bmw.t.u-tokyo.ac.jp (S.K.); yoshinaga@bmw.t.u-tokyo.ac.jp (N.Y.); 2 Innovation Center of NanoMedicine (iCONM), Kawasaki Institute of Industrial Promotion, 3-25-14 Tonomachi, Kawasaki-ku, Kawasaki 210-0821, Japan; 3 Department of Otorhinolaryngology and Head and Neck Surgery, Graduate School of Medicine and Faculty of Medicine, The University of Tokyo, 7-3-1 Hongo, Bunkyo-ku, Tokyo 113-0033, Japan; tempesta.50.1@gmail.com (K.I.); yumatsumoto@mac.com (Y.M.)

**Keywords:** mRNA delivery, mRNA-engineering, lipid nanoparticles, polyethylene glycol, hybridization, RNA oligonucleotide, steric stabilization

## Abstract

Lipid nanoparticles (LNPs) exhibit high potential as carriers of messenger RNA (mRNA). However, the arduous preparation process of mRNA-loaded LNPs remains a huge obstacle for their widespread clinical application. Herein, we tackled this issue by mRNA PEGylation through hybridization with polyethylene glycol (PEG)-conjugated RNA oligonucleotides (PEG-OligoRNAs). Importantly, mRNA translational activity was preserved even after hybridization of 20 PEG-OligoRNAs per mRNA. The straightforward mixing of the PEGylated mRNA with lipofectamine LTX, a commercial lipid-based carrier, just by pipetting in aqueous solution, allowed the successful preparation of mRNA-loaded LNPs with a diameter below 100 nm, whereas the use of non-PEGylated mRNA provided large aggregates above 100- and 1000-nm. In vivo, LNPs prepared from PEG-OligoRNA-hybridized mRNA exhibited high structural stability in biological milieu, without forming detectable aggregates in mouse blood after intravenous injection. In contrast, LNPs from non-PEGylated mRNA formed several micrometer-sized aggregates in blood, leading to rapid clearance from blood circulation and deposition of the aggregates in lung capillaries. Our strategy of mRNA PEGylation was also versatile to prevent aggregation of another type of mRNA-loaded LNP, DOTAP/Chol liposomes. Together, our approach provides a simple and robust preparation method to LNPs for in vivo application.

## 1. Introduction

Messenger RNA (mRNA) encoding therapeutic proteins has recently demonstrated its potential to treat a variety of diseases in preclinical and clinical studies [1,2]. For in vivo mRNA delivery, lipid nanoparticles (LNPs) are widely used to prevent mRNA degradation before translating proteins in target cells, and exhibit promising outcome especially in mRNA delivery to the liver and the spleen, owing to their intrinsic tropism to these organs [3,4,5]. On the other hand, a critical issue for the widespread clinical application of LNPs is the complicated procedures for their formulation. The main reason for such intricate processes is the uncontrollable aggregation of cationic lipids with large and highly charged anionic molecules, such mRNA, resulting after directly mixing these components, as extensively demonstrated for LNPs loading plasmid DNA (pDNA) [6]. Thus, the preparation of uniform nanometer-sized mRNA-loaded LNPs often requires special devices or techniques, such as microfluidics, for mixing the lipid components and mRNA, which allows controlling the size of LNPs [7,8] and replacing organic solvents with buffers for biological usage [9,10]. On the other hand, the development of a simple and scalable technique capable of preventing such aggregate formation in buffer would be highly valuable for the straightforward formulation of clinically relevant mRNA-loaded LNPs. 

Herein, we proposed a simple yet robust and versatile strategy of LNP preparation by installing polyethylene glycol (PEG) onto the mRNA molecules. The steric repulsive forces between PEG chains are expected to prevent LNP aggregation after mixing parent LNPs with mRNA. Besides the undemanding assembly, this strategy would allow easy preparation of mRNA-loaded LNPs in bedside just by mixing two aqueous solutions of LNPs and mRNA, without the need of special devices and buffer replacement, as well as eliminating long-term storage of mRNA-loaded LNPs in aqueous solution, which is often challenging. Moreover, the LNPs resulting from the proposed method are PEGylated, which contributes to LNP steric stabilization under biological milieu [11,12]. In addition, the PEG density on LNPs can be easily tuned by changing the number of PEG chains installed onto mRNA molecules. Therefore, once the parent LNPs are established, this strategy would allow easy optimization of PEG density on LNPs to obtain desirable biodistribution after in vivo administration. 

For pursuing the proposed approach, the PEGylation of mRNA without affecting its translation activity is a critical issue. While chemically modified bases, such as 5-methylcytosine, and N^1^-methyl-pseudouracil, are widely used for manipulating mRNA chemical structures [13,14,15], even subtle base modification could lead to drastic decrease in translation efficiency, depending on the formulations of modification [16,17]. We have recently developed an mRNA-engineering strategy based on the installation of functional moieties to mRNA through hybridization of the mRNA with short RNA oligonucleotides [18,19]. While double stranded RNA (dsRNA) structures prepared in this strategy can also result in the inhibition of mRNA translation, as well as the induction of inflammatory responses [20,21], these undesirable outcomes were effectively avoided by shortening hybridization lengths to 17 nt. Thus, based on these studies, we hybridized mRNA with PEGylated 17 nt RNA oligonucleotides (PEG-OligoRNAs) for mRNA PEGylation in this study. Using a commercially-available reagent, lipofectamine LTX, and a widely-used liposomal formulation, 1,2-bis(dioleoyloxy)-3-(trimethylamonio)propane (DOTAP)/cholesterol (Chol), as model LNPs to load mRNA hybridized with PEG-OligoRNAs (PEG-OligoRNAs/mRNA), we studied the feasibility of our strategy through detailed physicochemical characterization and functional analyses using cultured cells and mice.

## 2. Results

### 2.1. Hybridization of mRNA with PEG-OligoRNAs

**Figure 1:**
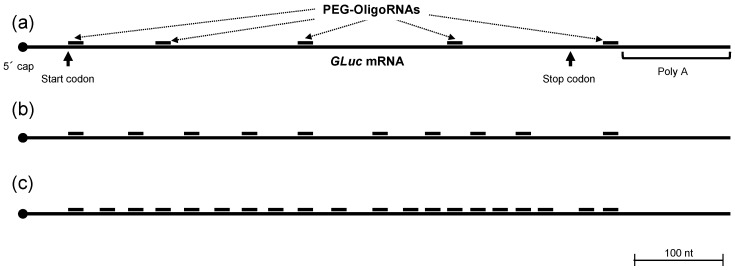
Positions in *GLuc* mRNA to which each PEG-OligoRNA hybridizes. (**a**) 5 PEG-OligoRNAs/mRNA. (**b**) 10 PEG-OligoRNAs/mRNA. (c) 20 PEG-OligoRNAs/mRNA. A scale bar shows the length of 100 nt.

RNA oligonucleotides were designed to have a 12 kDa PEG chain on the 5′ end of a 17 nt hybridization sequence. We prepared mRNA hybridized with 5, 10 and 20 different PEG-OligoRNAs. The PEG-OligoRNAs were designed to target the positions throughout 5′ to 3′ ends of 783 nt *Gaussia luciferase* (*GLuc*) mRNA (Figure 1). In the selection of target sequences in *GLuc* mRNA, dsRNA region was avoided (Supplementary Table S1), by using a software that predicts RNA secondary structure [22], unless no unstructured regions were found nearby, because endogenous base-paring in mRNA might hamper the hybridization of PEG-OligoRNAs to the mRNA. The mRNA was hybridized with PEG-OligoRNAs with molar ratio of each sequence of PEG-OligoRNA to mRNA of 1:1. Then, gel permeation chromatography (GPC) was performed to measure hybridization efficiency. The peak intensity derived from unhybridized free PEG-OligoRNAs was very weak, revealing high hybridization efficiency of 87–95% in every tested formulation (Table 1, Supplementary Figure S1).

### 2.2. Translation Activity of mRNA after Hybridization with PEG-OligoRNAs

**Figure 2:**
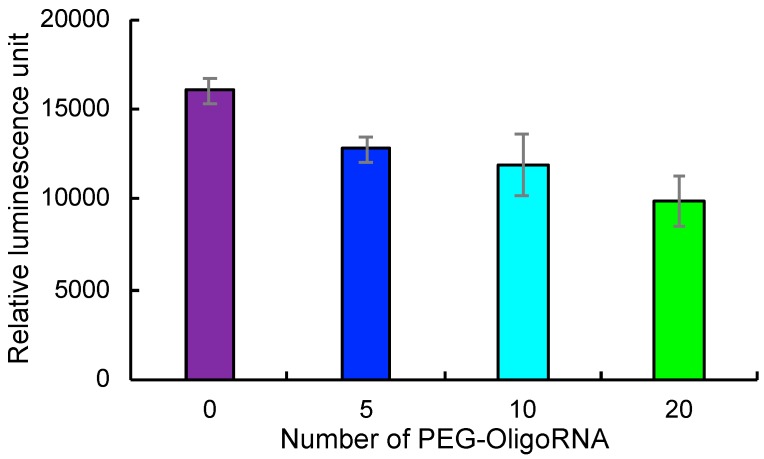
Translation efficiency from mRNA in cell free system. *GLuc* mRNA hybridized with PEG-OligoRNAs was incubated in rabbit reticulocyte lysate. After 4 h of incubation, GLuc protein expression levels were measured. *n* = 3. Data are presented as mean ± standard error of the mean.

Translation activity of PEG-OligoRNAs/mRNA was measured in cell free translational system composed of rabbit reticulocyte lysate. Luciferase protein expression levels decreased with the increase in PEG-OligoRNA numbers hybridizing to mRNA (Figure 2). However, more than 60% of translational activity was maintained even after hybridizing 20 PEG-OligoRNAs, compared to unhybridized mRNA. This result is consistent with our previous report, showing that mRNA hybridized with a large number of cholesterol-installed RNA oligonucleotides preserved its translational activity [19].

### 2.3. Characterization of Lipofectamine LTX-based LNPs Loading PEG-OligoRNAs/mRNA

PEG-OligoRNAs/mRNA was then mixed with lipofectamine LTX, a commercially available lipid-based transfection reagent, just by pipetting in aqueous solution. Two LNP formulations were prepared by mixing mRNA with minimal or maximal amount of lipofectamine LTX solution that the manufacturer suggests to use. The resulting LNPs are designated as Lipo-Min and Lipo-Max, respectively. Gel electrophoresis of the LNPs was performed to check successful loading of PEG-OligoRNAs/mRNA to the LNPs. Bands corresponding to free PEG-OligoRNAs/mRNA were undetectable in every tested formulation, demonstrating that almost all PEG-OligoRNAs/mRNA was associated with lipofectamine LTX reagent both in Lipo-Min and Lipo-Max (Supplementary Figure S2).

**Figure 3:**
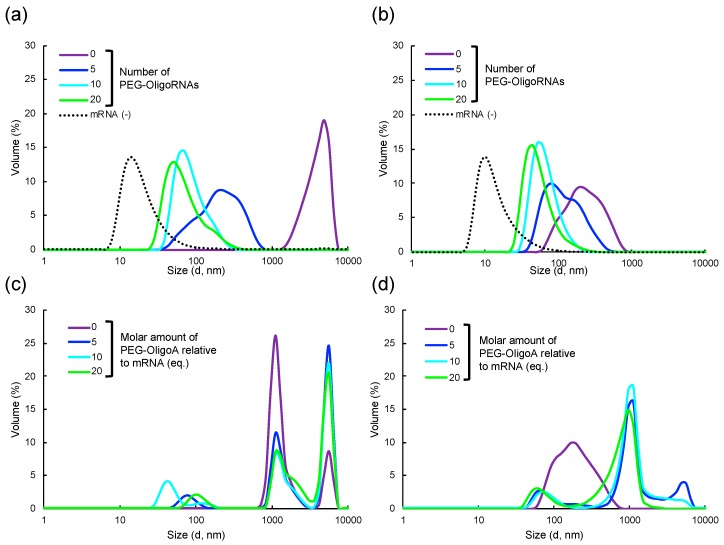
Dynamic light scattering measurement of Lipofectamine LTX/mRNA LNPs. Two LNP formulations were prepared by mixing mRNA with (**a**,**c**) minimal or (**b**,**d**) maximal volume of lipofectamine LTX solution that the manufactures suggest to use ((**a**,**c**) Lipo-Min and (**b**,**d**) Lipo-Max). (**a**,**b**) The size of the LNP without mRNA addition (mRNA (−)), and that loading mRNA hybridized with 0, 5, 10, or 20 PEG-OligoRNAs was measured. (**c**,**d**) PEG-OligoA was used as a control PEG-OligoRNA that does not hybridize to mRNA. 5 eq., 10 eq., and 20 eq. of PEG-OligoA relative to mRNA was added to mRNA for preparation of Lipo-Min and Lipo-Max.

The sizes of the LNPs loaded with mRNA were measured using dynamic light scattering (DLS). The addition of unhybridized mRNA to the parent Lipofectamine LTX shifted the size from around 50 nm up to 5000 nm when we used Lipo-Min, and to several hundred nanometers when we used Lipo-Max (Figure 3a,b, Table 2). Hybridization of PEG-OligoRNAs was effective in preventing aggregate formation in a manner dependent on the number of PEG-OligoRNAs. Eventually, Lipo-Min and Lipo-Max loading 20 PEG-OligoRNA/mRNA exhibited sizes of 78 nm and 57 nm with narrow size distribution, respectively. Notably, LNP size should be ideally below 200 nm to obtain prolonged blood circulation with escaping the uptake by reticuloendothelial system (RES) [23,24]. Moreover, size control in the sub-100 nm range is important for achieving deep and broad distribution in some tissues, such as fibrotic cancer tissues [25].

To see whether the hybridization process is required to prevent aggregation using PEG-OligoRNAs, we used PEGylated 17 nt oligoadenine (PEG-OligoA), as a control PEG-OligoRNA that does not hybridize to mRNA. Lipo-Min and Lipo-Max were prepared from the mixture of mRNA and PEG-OligoA for DLS measurement. The addition of PEG-OligoA failed to inhibit LNP aggregation in both Lipo-Min and Lipo-Max, even at the same molar amount of PEG-OligoA as that of PEG-OligoRNAs used to prepare 20 PEG-OligoRNAs/mRNA (Figure 3c,d). This result demonstrates that PEG-OligoRNAs should be hybridized to mRNA to prevent LNP aggregation.

Interestingly, in Lipo-Max, the addition of mRNA and PEG-OligoA to Lipofectamine LTX solution resulted in the preparation of large particles with the size of 1,000 nm or even larger (Figure 3d), while the size of Lipo-Max loaded with unhybridized mRNA was originally several hundred nanometers (Figure 3b). Thus, the presence of PEG-OligoA prompted the aggregation of Lipo-Max. However, addition of PEG-OligoA to the parent Lipofectamine LTX solution without the presence of mRNA failed to induce such aggregation (Supplementary Figure S3). Based on these observations, it is reasonable to assume that the binding of PEG-OligoA to Lipofectamine LTX causes the change in binding behavior of non-PEGylated mRNA to Lipofectamine LTX, which then results in LNP aggregation.

**Figure 4:**
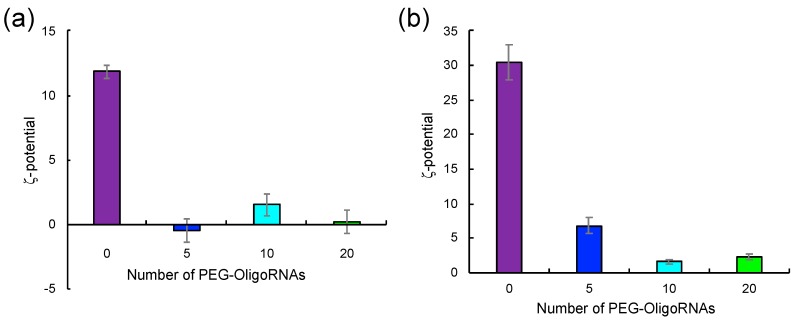
ζ-potential measurement of Lipofectamine LTX/mRNA LNPs. Two LNP formulations were prepared by mixing mRNA with (**a**) minimal or (**b**) maximal volume of lipofectamine LTX solution that the manufactures suggest to use ((**a**) Lipo-Min and (**b**) Lipo-Max). ζ-potential of the LNPs without mRNA addition (mRNA (−)), and those loading mRNA hybridized with 0, 5, 10, or 20 PEG-OligoRNAs were measured. *n* = 6. Data are presented as mean ± standard error of the mean.

Then, LNP ζ-potential was measured to examine the degree of LNP PEGylation, which may influence the ζ-potential. Lipo-Min and Lipo-Max loaded with unhybridized mRNA exhibited high ζ-potential of +12 mV and +31 mV, respectively (Figure 4). The ζ-potential decreased after hybridization of mRNA with PEG-OligoRNAs, and the drop correlated with the number of PEG-OligoRNAs hybridizing to mRNA. Lipo-Min and Lipo-Max loading 20 PEG-OligoRNA showed almost neutral ζ-potential of +0.2 mV and +2.3 mV, respectively (Figure 4). Thus, the cationic surface charge of Lipofectamine LTX/mRNA LNPs was masked after hybridization of mRNA with PEG-OligoRNAs, indicating the successful PEGylation of the LNPs.

### 2.4. Transmission Electron Microscopic Observation of Lipofectamine LTX-based LNPs

**Figure 5:**
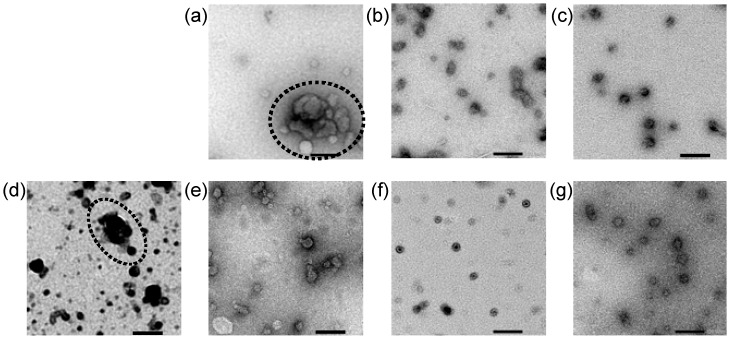
Transmission electron microscopic imaging of Lipofectamine LTX/mRNA LNPs. Two LNP formulations were prepared by mixing mRNA with (**a**–**c**) minimal or (**d**–**g**) maximal volume of lipofectamine LTX solution that the manufactures suggest to use ((**a**–**c**) Lipo-Min and (**d**–**g**) Lipo-Max). (**a**–**c**) Lipo-Min loaded with (**a**) 5 PEG-OligoRNAs/mRNA, (**b**) 10 PEG-OligoRNAs/mRNA, and (**c**) 20 PEG-OligoRNAs/mRNA. (**d**–**g**) Lipo-Max loaded with (**d**) unhybridized mRNA, (**e**) 5 PEG-OligoRNAs/mRNA, (**f**) 10 PEG-OligoRNAs/mRNA, and (**g**) 20 PEG-OligoRNAs/mRNA. Dotted circle showed large particles prepared through association of several smaller particles. Scale bars: 100 nm.

Direct observation of Lipo-Min and Lipo-Max was performed using transmission electron microscopy (TEM). Representative images of Lipo-Min loaded with 5–20 PEG-OligoRNAs/mRNA and Lipo-Max loaded with 0–20 PEG-OligoRNAs/mRNA are shown in Figure 5, while aggregation hampered TEM observation of Lipo-Min loaded with unhybridized mRNA (see Figure 3a). Images of Lipo-Min loaded with 5 PEG-OligoRNAs/mRNA and Lipo-Max loaded with unhybridized mRNA revealed the formation of large LNPs with the size over 100 nm, which were prepared through association of several smaller particles (see dotted circle in Figure 5a,d). In accordance with this observation, a previous research reported that addition of pDNA to cationic LNP induced the aggregation of the LNP by crosslinking of several LNPs through pDNA strands [6]. Thus, aggregation of LNPs loading non-PEGylated mRNA or mRNA hybridized with a small number of PEG-OligoRNAs may be explained by crosslinking of several LNPs through mRNA strands. In contrast, mRNA hybridization with a large number of PEG-OligoRNAs leads to LNP coating with high density of PEG chains, as indicated by ζ-potential neutralization of LNP (Figure 4), which may inhibit the association between LNPs, thereby preventing the LNP aggregation (Figure 5b,c,f,g).

### 2.5. Loading of PEG-OligoRNA/mRNA to DOTAP/Chol liposome

**Figure 6:**
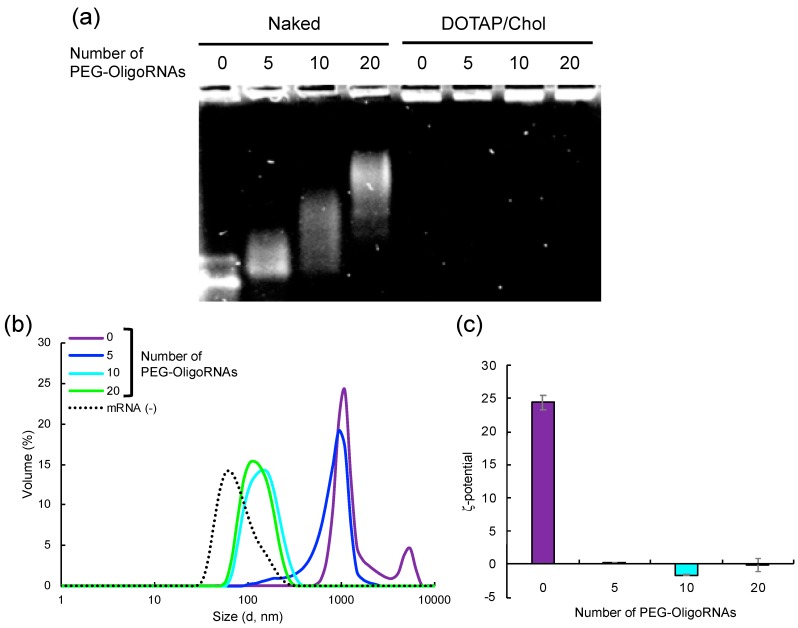
Characterization of DOTAP/Chol LNPs loaded with PEG-OligoRNAs/mRNA. (**a**) Gel electrophoresis of unhybridized mRNA and mRNA hybridized with 5, 10, or 20 PEG-OligoRNAs, in a naked form or after complexation with DOTAP/Chol liposomes. (**b**) The sizes and (**c**) ζ-potential of the DOTAP/Chol LNPs without mRNA addition (mRNA (−)), and that loading mRNA hybridized with 0, 5, 10, or 20 PEG-OligoRNAs were measured. *n* = 6 for (**c**). Data are presented as mean ± standard error of the mean.

To investigate the versatility of our approach, PEG-OligoRNAs/mRNA was then merged with DOTAP/Chol liposome. Two aqueous solutions of PEG-OligoRNAs/mRNA and DOTAP/Chol liposome were mixed just by pipetting, at amine groups in DOTAP to phosphate groups in PEG-OligoRNAs/mRNA (N/P) ratio of 3. Gel electrophoresis experiment confirmed successful association of almost all PEG-OligoRNAs/mRNA to the liposomes in all tested formulations (Figure 6a). In DLS measurement, addition of unhybridized mRNA led to the formation of two types of aggregates with the size around 1000 nm and 5000 nm, respectively (Figure 6b, Table 3). Hybridization of PEG-OligoRNAs was effective in preventing aggregate formation, and the ability to prevent the aggregation correlated with the number of PEG-OligoRNAs. Eventually, LNPs loaded with 20 PEG-OligoRNA/mRNA exhibited the average size of 132 nm with narrow size distribution. In the ζ-potential measurement, LNPs loaded with unhybridized mRNA had high cationic charge of +24 mV. The cationic charge was successfully masked after hybridization of mRNA with 5-20 PEG-OligoRNAs, indicating the successful PEGylation of the LNPs (Figure 6c). Together, our approach of PEG-OligoRNA hybridization allowed easy preparation of PEGylated DOTAP/Chol-based LNPs loaded with mRNA with relatively small sizes and almost neutral surface charges, as is the case of Lipofectamine LTX-based LNPs (Figure 3, Figure 4 and Figure 5).

### 2.6. Introduction of Lipofectamine LTX-Based LNPs to Cultured Cells

**Figure 7:**
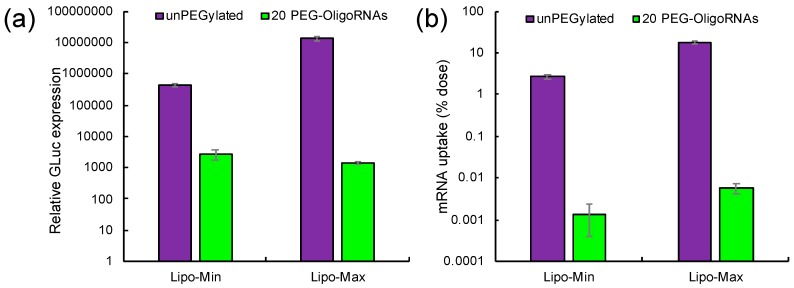
Introduction of Lipofectamine LTX/mRNA LNPs to cultured cells. Two LNP formulations were prepared by mixing *GLuc* mRNA with minimal or maximal volume of lipofectamine LTX solution that the manufactures suggest to use (Lipo-Min and Lipo-Max). LNP loaded with non-PEGylated mRNA and that loaded with 20 PEG-OligoRNAs/mRNA were transfected to HuH-7 cells. (**a**) GLuc protein concentration in the culture medium 4 h after the transfection. *n* = 6. (**b**) *GLuc* mRNA uptake to HuH-7 cells measured using qRT-PCR 4 h after the transfection. *n* = 4. Data are presented as mean ± standard error of the mean.

Biological function of Lipofectamine LTX-based LNPs loading 20 PEG-OligoRNAs/mRNA was then investigated in vitro using HuH-7 cells. First, mRNA introduction efficiency was evaluated using *GLuc* mRNA as a reporter. LNP loaded with non-PEGylated mRNA exhibited 2–4 orders higher efficiency of GLuc protein expression compared to that loaded with 20 PEG-OligoRNAs/mRNA (Figure 7a). To study the mechanism, efficiency of mRNA cellular uptake was evaluated by measuring *GLuc* mRNA amount in the cells through quantitative real-time PCR (qRT-PCR). The efficiency of mRNA uptake decreased by 3–4 orders after hybridization of 20 PEG-OligoRNAs compared to LNP loaded with unhybridized mRNA (Figure 7b), showing the profile consistent to the measurement of GLuc expression. Thus, the translational efficiency standardized with the amount of mRNA taken up by cells was comparable regardless of the hybridization of 20 PEG-OligoRNAs. This result suggests that the decrease in GLuc protein expression efficiency after LNP PEGylation is largely attributed to limited cellular uptake of the LNPs, rather than reduced translational activity of mRNA. Indeed, in cell free translational system, hybridization of PEG-OligoRNAs induced minimal effect on mRNA translational activity (Figure 2).

### 2.7. In Vivo Behavior of Lipofectamine LTX-Based LNPs after Systemic Delivery

**Figure 8:**
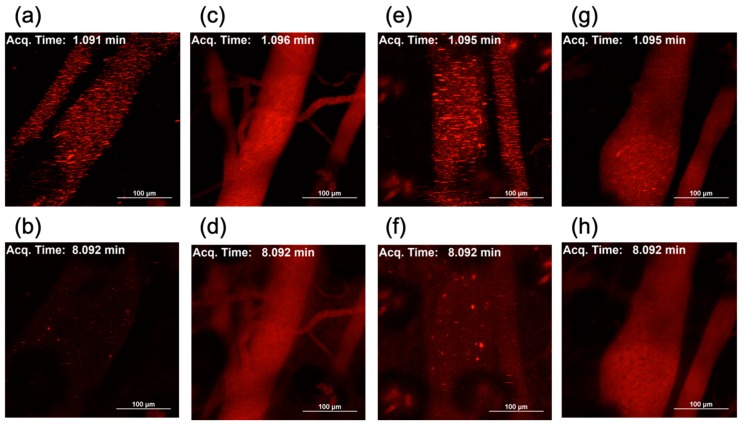
Intravital real-time confocal observation of Lipofectamine LTX-based LNPs in the blood. Using *GLuc* mRNA labeled with Cy5, two LNP formulations were prepared by mixing the mRNA with minimal or maximal volume of lipofectamine LTX solution that the manufacture suggests to use ((**a**–**d**) Lipo-Min and (**e**–**h**) Lipo-Max). Then, the LNPs was intravenously injected to the mice for observation of the blood vessel in the earlobes using intravital confocal microscopy. (**a**,**c**,**e**,**g**) Images 1 min after the injection. (**b**,**d**,**f**,**h**) Images 8 min after the injection. (**a**,**b**) Lipo-Min loaded with non-PEGylated mRNA. (**c**,**d**) Lipo-Min loaded with 20 PEG-OligoRNAs/mRNA. (**e**,**f**) Lipo-Max loaded with non-PEGylated mRNA. (**g**,**h**) Lipo-Max loaded with 20 PEG-OligoRNAs/mRNA.

Lastly, the in vivo behavior of the Lipofectamine LTX-based LNPs was evaluated after their intravenous injection into mice. Real-time behavior of LNPs loaded with Cy5-labeled mRNA in the blood circulation was observed using intravital confocal microscopy. In both cases of Lipo-Min and Lipo-Max loaded with non-PEGylated mRNA, several micrometer-sized aggregates were observed 1 min after the injection (Figure 8a,e). Note that minimal detectable size of the aggregate is theoretically estimated to be above 700 nm, based on the pixel size of 620 nm × 620 nm, and on the Cy5 detection wavelength of 700 nm. The fluorescence signal in the blood was rapidly decreased, and almost no signal was detected as early as 8 min after the injection (Figure 8b,f, Videos S1 and S3), revealing rapid clearance of non-PEGylated Lipo-Min and Lipo-Max. In contrast, when 20 PEG-OligoRNAs/mRNA was loaded to Lipo-Min and Lipo-Max, the fluorescence signal in the blood was uniform without detectable aggregation throughout the observation periods, and high fluorescence intensity was observed in the blood even 8 min or later after the injection (Figure 8c,d,g,h, Videos S2 and S4). This observation indicates effective steric stabilization of Lipo-Min and Lipo-Max by mRNA PEGylation also in the biological milieu, which resulted in prolonged blood circulations.

**Figure 9:**
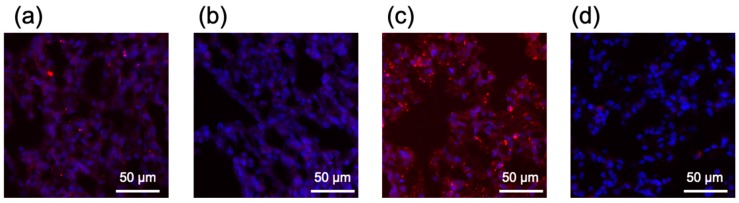
Distribution of Lipofectamine LTX-based LNPs in the lung. Using *GLuc* mRNA labeled with Cy5, two LNP formulations were prepared by mixing the mRNA with minimal or maximal volume of lipofectamine LTX solution that the manufacture suggests to use ((**a**,**b**) Lipo-Min and (**c**,**d**) Lipo-Max). Then, the LNPs were intravenously injected to the mice. Five min after the injection, the lung was excised for preparation of frozen tissue sections. Blue: nucleus (DAPI). (**a**) Lipo-Min loaded with non-PEGylated mRNA. (**b**) Lipo-Min loaded with 20 PEG-OligoRNAs/mRNA. (**c**) Lipo-Max loaded with non-PEGylated mRNA. (**d**) Lipo-Max loaded with 20 PEG-OligoRNAs/mRNA.

According to a previous report, the aggregation of nucleic acid complexes in the blood induced lung embolism, even causing the death of experimental animals [26]. Then, we observed the distribution of Lipo-Min and Lipo-Max in the tissue section of the lung after injection of Lipo-Min and Lipo-Max loaded with Cy5-labeled mRNA. For Lipo-Min and Lipo-Max loaded with non-PEGylated mRNA, several micrometer-sized red dots were observed throughout the lung tissue 5 min after injection, which presumably showed deposition of aggregates in the lung capillary (Figure 9a,c). On the other hand, such deposition of LNP aggregates in the lung was effectively prevented after hybridization of 20 PEG-OligoRNAs to mRNA (Figure 9b,d). Similarly, several-micrometer-sized aggregates from unPEGylated Lipo-Max were deposited also in the spleen and the liver, while Lipo-Max from 20 PEG-OligoRNAs/mRNA showed reduced accumulation to these organs (Supplementary Figure S4). These observations are consistent with the real-time LNP imaging in blood circulation, demonstrating the effectiveness of PEG-OligoRNA hybridization for inhibiting the LNP aggregation in blood (Figure 8). With avoidance of tissue deposition and clearance mechanism, the PEGylated LNP showed prolonged blood circulation compared to non-PEGylated LNPs as shown in Figure 8.

## 3. Discussion

LNP loading of mRNA requires complicated procedures, which hamper the widespread clinical application of the mRNA LNPs. In this study, we developed a simple, robust and versatile procedure for mRNA LNP preparation by mRNA PEGylation. Using this strategy, mRNA-loaded LNPs with regulated sizes and close-to-neutral surface charges were prepared just by mixing two aqueous solutions of parent LNP and mRNA without the need of buffer exchange for subsequent biological usage (Figure 3, Figure 4, Figure 5 and Figure 6). Such easy procedure allows LNP preparation even at bedside, which additionally provides a solution to the issue of mRNA storage. Although lyophilization is an attractive approach for long-term storage of mRNA without degradation [27], lyophilization of mRNA-loaded LNPs could influence their physicochemical properties. On the other hand, lyophilization of mRNA molecule is an established technique, allowing long-term mRNA storage even at room temperature [28]. Our strategy would allow preparation of mRNA-loaded LNPs immediately after rehydration of lyophilized mRNA, minimizing time of mRNA storage in aqueous solution.

For mRNA PEGylation, 17 nt PEG-OligoRNAs were hybridized to mRNA. Importantly, addition of PEG-OligoA, an unhybridizing control, failed to prevent LNP aggregation (Figure 3c,d), demonstrating the importance of mRNA PEGylation through hybridization of PEG-OligoRNAs. Moreover, hybridization of 20 PEG-OligoRNAs per mRNA induced minimal influence on translational activity of mRNA (Figure 2). This result is consistent with our previous report of mRNA-engineering, showing that short RNA oligonucleotides possessing functional moieties can be hybridized to mRNA with preserved translational activities [19]. Cellular mechanisms of unwinding dsRNA in an ATP-consuming manner presumably contributed to efficient translation from mRNA hybridized with short RNA oligonucleotides [29].

LNP PEGylation showed large influence on in vivo blood circulation profile after systemic injection (Figure 8). LNPs loaded with non-PEGylated mRNA formed aggregates immediately after the injection, leading to rapid clearance of the LNPs from the blood circulation. Notably, the non-PEGylated LNPs were deposited in the lung tissues, indicating the occlusion of lung capillary by the aggregates. Importantly, such lung embolism can cause the death of mice [26]. On the other hand, LNP PEGylation through PEG-OligoRNA hybridization to mRNA is effective in preventing LNP aggregation in the blood, and LNP deposition in the lung, demonstrating high structural stability of the LNPs in biological milieu and their improved safety for in vivo usage. Such structural stabilization after PEGylation may be explained by PEG steric repulsive force preventing the association of LNP with surrounding proteins, blood cells or LNPs in the blood [30].

The mRNA introduction efficiency to the cultured cells decreased after LNP PEGylation, which may be explained by PEG steric repulsive force inhibiting the non-specific interaction of LNPs to cells and LNP uptake to the cells (Figure 7). The PEGylation of the LNPs can also induce inhibitory effect on intracellular processes, such as endosomal escape [31]. Several strategies were reported for solving these issues of PEGylation, so called “PEG dilemma”, and facilitating cellular uptake and endosomal escape, including the installation of ligands to PEG, the optimization of surface PEG density and the selective cleavage of PEG in target tissues or cells [32,33,34,35,36]. We plan combinational use of these strategies with the mRNA PEGylation approach, to achieve efficient in vivo mRNA introduction in the future study.

The use of PEGylated lipids is an alternative strategy to prevent the aggregation after mRNA addition to LNPs, and feasibility of this strategy was well studied in LNP preparation for pDNA delivery. Although this strategy showed some effect on steric stabilization of LNP, PEGylated LNPs still aggregate after pDNA addition, depending on their formulations [37,38]. Moreover, mRNA-loaded LNPs have been prepared by using PEGylated lipids and organic solvents, leading to the assembly of particles with several hundred nanometer diameter and highly cationic ζ-potential [39]. Our strategy has potential to prepare sub-100 nm-sized LNPs with neutral surface charge, by mRNA PEGylation alone or by combination with PEGylated lipids. 

Our strategy can potentially be applied to polymer-based mRNA carriers, which provide promising alternatives to lipid-based carriers for targeting various tissues other than the liver and the spleen [40,41,42,43], while the liver and the spleen can be more efficiently targeted using LNPs due to their tropism to these organs. By using PEGylated mRNA, the complicated processes of PEG conjugation to polycation can be avoided. Furthermore, as is the case in LNPs, PEGylation of polyplexes is beneficial for their steric stabilization in biological milieu [35,44] and alleviation of mRNA immunogenicity [45]. 

In conclusion, we developed a simple, robust and versatile procedure of mRNA-loaded LNP preparation, providing a smart solution to so far complicated procedures. The PEGylation of mRNA allowed the preparation of size-controlled LNP by mixing two aqueous solutions of mRNA and parent LNP by using pipetting. Importantly, mRNA was successfully PEGylated with minimal influence on its translational activity by hybridizing mRNA with PEGylated short RNA oligonucleotides. The PEGylated LNP thus obtained exhibited high structural stability in blood after intravenous injection, while non-PEGylated LNP rapidly aggregated in blood, presumably causing lung embolism. By providing methods for easy preparation of mRNA LNP for in vivo usage, our approach has the potential to accelerate the widespread clinical use of existing and newly developed mRNA LNP formulations.

## 4. Materials and Methods 

### 4.1. Preparation of mRNA

mRNA was prepared as previously described [46]. Briefly, template DNA containing *Gaussia luciferase* (*GLuc*) coding sequence and poly 120 bp A/T under T7 promoter was constructed from a pSP73 plasmid (Promega, Madison, WI, USA) and a pCMV-Gluc control plasmid (New England BioLabs, Ipswich, MA, USA). In vitro transcription was performed using mMESSAGE mMACHINE T7 Ultra Kit (Ambion, Carlsbad, CA, USA), followed by mRNA purification using RNeasy Mini Kit (Qiagen, Hilden, Germany), and spectroscopic determination of its concentration using a NanoDrop 1000 spectrophotometer (NanoDrop Technologies Inc., Wilmington, DE, USA). On-chip capillary electrophoresis was performed using an Agilent2100 Bioanalyzer (Agilent, Santa Clara, CA, USA), after denaturing mRNA at 72 °C for 2 min, followed by rapid cooling of the mRNA solution on ice. The result demonstrated successful preparation of homogenous mRNA strands (Supplementary Figure S5).

### 4.2. Hybridization of mRNA with PEG-OligoRNAs

17 nt RNA oligonucleotides installed with PEG (M_W_: 12 KDa) at their 5′ end (PEG-OligoRNAs) were purchased from GeneDesign Inc. (Osaka, Japan). These PEG-OligoRNAs have sequences complementary to the target sequences in *GLuc* mRNA shown in Supplementary Table S1. The PEG-OligoRNAs were mixed with *GLuc* mRNA with molar ratio of each sequence of PEG-OligoRNA to mRNA of 1:1 to obtain final *GLuc* mRNA concentration of 100 ng/μL in the buffer containing 10 mM HEPES and 150 mM NaCl. Then, the RNA solution was kept at 65 °C for 5 min, followed by gradual cooling to 30 °C in 90 min.

### 4.3. Cell Free Translation

Cell free translation of *GLuc* mRNA was performed using Rabbit Reticulocyte Lysate System, Nuclease Treated (Promega). After 4 h of the incubation of *GLuc* mRNA (50 ng) in rabbit reticulocyte lysate (14 μL), GLuc expression level was measured using Renilla Luciferase Assay System (Promega) and Lumat^3^ LB9508 luminometer (Berthold Technologies, Bad Wildbad, Germany). 

### 4.4. Preparation of Lipofectamine LTX/mRNA LNPs

PEG-OligoRNAs/mRNA was mixed with LTX solution equipped in Lipofectamine LTX (Invitrogen, Carlsbad, CA, USA) at the ratio of 1 μg mRNA to 2 μL LTX solution for Lipo-Min, and that of 1 μg mRNA to 5 μL LTX solution for Lipo-Max. Final mRNA solution was adjusted to 33.3 ng/μL in the buffer containing 10 mM HEPES and 50 mM NaCl.

### 4.5. Characterization of LNPs

The LNP solutions thus obtained were used for characterization including gel electrophoresis, DLS, and ζ-potential measurement. After adding 20 μL of sample solution with 4 μL of 30% glycerol, gel electrophoresis was performed using 0.9% agarose gel at 100 mV for 35 min. After 1 h incubation in 0.5 mg/L ethidium bromide solution (NIPPON GENE Co., Ltd., Tokyo, Japan), the gel was imaged using Gel Doc ER system (BioRad, Hercules, CA, USA). Zetasizer Nano-ZS (Malvern Instruments, Worcestershire, UK) equipped with an Ar laser (λ = 532 nm) was used for DLS measurement with scattering angle of 173 ° at 25 °C. The diameter was calculated using Stokes Einstein equation. ζ-potential was measured through laser-doppler electrophoresis using Möbius ζ™ (Wyatt Technology Corporation, Santa Barbara, CA, USA) equipped with a 532 nm laser, followed by the calculation based on the Smoluchowski’s equation. All of these characterization experiments were performed at mRNA concentration of 33.3 ng/μL in the buffer containing 10 mM HEPES and 50 mM NaCl.

### 4.6. Transmission Electron Microscopic Observation

An Eiko IB-3 ion coater (Eiko Engineering Co. Ltd., Tokyo, Japan) was used to hydrophilize carbon-coated copper TEM grids (Cu400, JEOL Ltd., Tokyo, Japan) by glow discharge. On the grid, 2 μL of LNP solution loaded with 20 ng of PEG-OligoRNAs/mRNA was mixed with 2 μL of 2% *w*/*v* uranyl acetate solution. After 30 s, the droplet was removed using a filter paper and then the grid was dried. The sample was observed using a JEM-1400 (JEOL Ltd.) at acceleration voltage of 120 kV.

### 4.7. Preparation of DOTAP/Chol LNPs

Parent DOTAP/Chol liposomes were prepared according to a previous report, with modification [47]. DOTAP (Avanti Polar Lipids, Alabaster, AL, USA) and cholesterol (Wako Pure Chemical Industrial Ltd., Osaka, Japan) was dissolved in chloroform, at DOTAP to Chol molar ratio 10:9. The solvent was evaporated under an Argon gas stream to form thin lipid film. After resuspension with nuclease free water and overnight incubation at room temperature, the sample was sonicated for 5 min, followed by sequential extrusion through filters with pore sizes of 400 nm, 100 nm, and 50 nm using Avanti Mini Extruder (Avanti Polar Lipids). Then, the parent liposomes were mixed with PEG-OligoRNAs/mRNA at amine groups in DOTAP to phosphate groups in PEG-OligoRNAs/mRNA (N/P) ratio of 3. After adjusting mRNA concentration to 10 ng/μL in the buffer containing 10 mM HEPES and 15 mM NaCl, characterization of the LNP was performed as described in the Section 4.5.

### 4.8. mRNA Introduction to Cultured Cells

HuH-7 cells (RIKEN cell bank) were cultured using Dulbecco’s modified Eagle’s medium (DMEM) (Sigma-Aldrich Co., Madison, WI, USA) containing 10% fetal bovine serum (FBS) (GE Healthcare, Chicago, IL, USA) and 1% penicillin/streptomycin (Sigma–Aldrich) in a humidified atmosphere with 5% CO_2_ at 37 °C. For GLuc protein expression assay, the cells were seeded onto 96 well plate at the density of 10,000 cells/well. After 24 h of the incubation, the cells were washed with PBS, and added with culture medium. Then, Lipo-Min and Lipo-Max loading 100 ng of mRNA were added to each well. After 4 h of the incubation, 10 μL of culture medium was collected for luciferase assay, performed as described in the Section 4.3. For qRT-PCR-based evaluation of mRNA cellular uptake, HuH-7 cells were seeded onto 24 well plate at the density of 20,000 cells/well and incubated for 24 h. After washing cells with PBS, and replacing culture medium, Lipo-Min and Lipo-Max loading 1 μg of mRNA were added to each well. After 4 h incubation, cells were washed with PBS twice, and lysed with 1% 2-mercaptoethanol in RLT buffer equipped in RNeasy Mini Kit for 15 min at 37 °C. Then, the lysate was incubated for 5 min at 65 °C for RNA denaturing, followed by total RNA extraction using RNeasy Mini Kit. After reverse transcription using a ReverTra Ace qPCR RT Master Mix kit (Toyobo Life Science, Osaka, Japan), qRT-PCR was performed using a primer pair for *GLuc* mRNA (Forward; TGAGATTCCTGGGTTCAAGG, and Reverse; GTCAGAACACTGCACG TTGG), SYBR green master mix (Roche, Basel, Switzerland), and Mic qPCR cycler (Bio Molecular Systems, Queensland, Australia). 

### 4.9. Systemic Injection of LNPs to Mice

For in vivo observation, LNPs were prepared from mRNA labeled with Cy5, using Label IT Cy5 Labeling Kit (Mirus Bio Corporation, Madison, WI, USA). For real-time observation of LNPs in mouse blood, BALB/c mice (female, 7 weeks old, Charles River Laboratories, Yokohama, Japan) were anesthetized continuously with isoflurane and placed under a Nikon A1R confocal laser scanning microscope system attached to an upright ECLIPSE Ni-E equipped with an Apo LWD 20x water immersion objective. Lipo-Min or Lipo-Max loading 14 μg of Cy5-labeled mRNA were injected from the tail vein. Cy5 signals of mRNA were detected from the right ear lobe vessels using 640/700 nm excitation/emission filters. Recording of images were started 10 seconds before injection, continued for 3 min, followed by repeated recording with an interval of 5 min. For preparing tissue sections, mice were perfused with PBS and 4% paraformaldehyde 5 min after the LNP injection to BALB/c mice (female, 7 weeks old). The lung was incubated in 4% paraformaldehyde in PBS buffer overnight, in 10% sucrose in PBS buffer for 4 h, in 15% sucrose in PBS buffer for 4 h, and in 20% sucrose in PBS buffer for overnight, and then frozen in optimal cutting temperature (O. C. T.) compound (Sakura Finetek, Torrance, CA, USA). Sections (10-μm thick) were prepared, and mounted using Prolong Gold Antifade Mountant with DAPI (Thermo Fisher Scientific, Waltham, MA, USA). The sections were observed under the fluorescence microscope (BX-X800, Keyence, Osaka, Japan) using 20x objective lens. All animal experiments were conducted in accordance with the approval of the Animal Care and Use Committee of The University of Tokyo (Tokyo, Japan) (approval code: P18-034) and the Innovation Center of NanoMedicine, Kawasaki Institute of Industrial Promotion (Kanagawa, Japan) (approval code: A16-004-9).

## Figures and Tables

**Table 1:** Hybridization efficiency determined by HPLC.

Number of PEG-OligoRNAs	5	10	20
Hybridization efficiency (%)	87	95	94

**Table 2:** Dynamic light scattering measurement of Lipofectamine LTX/mRNA lipoplexes.

	Lipo-Min					Lipo-Max				
	mRNA (−)	Number of PEG-OligoRNAs				mRNA (−)	Number of PEG-OligoRNAs			
		0	5	10	20		0	5	10	20
Size (nm) ^a^	43	4071	234	90	78	25	254	132	68	57
PDI ^b^	0.42	0.26	0.19	0.13	0.21	0.45	0.20	0.19	0.12	0.18

^a^ Volume averaged size. ^b^ Polydispersity index.

**Table 3:** Dynamic light scattering measurement of DOTAP/Chol LNPs.

	mRNA (−)	Number of PEG-OligoRNAs			
		0	5	10	20
Size (nm) ^a^	84	1698	853	152	132
PDI ^b^	0.15	0.31	0.47	0.10	0.08

^a^ Volume averaged size. ^b^ Polydispersity index.

## Supplementary Materials

The following are available online, Figure S1: Gel permeation chromatography (GPC) for evaluation of hybridization efficiency, Figure S2: Gel electrophoresis of lipofectamine LTX/mRNA LNPs, Figure S3: Dynamic light scattering (DLS) measurement of Lipofectamine LTX added with PEG-OligoA, Figure S4: Distribution of Lipo-Max in the spleen and the liver, Figure S5: Capillary electrophoresis of *GLuc* mRNA, Table S1: Secondary structure in *GLuc* mRNA sequence, Video S1: Intravital real-time confocal observation of Lipo-Min loaded with non-PEGylated mRNA, Video S2: Intravital real-time confocal observation of Lipo-Min loaded with 20 PEG-OligoRNAs/mRNA, Video S3: Intravital real-time confocal observation of Lipo-Max loaded with non-PEGylated mRNA, Video S4: Intravital real-time confocal observation of Lipo-Max loaded with 20 PEG-OligoRNAs/mRNA.
