# Supplementary material for: PEG-OligoRNA Hybridization of mRNA for Developing Sterically Stable Lipid Nanoparticles toward In Vivo Administration
Source: Molecules. 2019 Apr 3;24(7):1303. doi: 10.3390/molecules24071303 (PMC6479949; doi:10.3390/molecules24071303)
Supplement: Supplementary file 1 [file molecules-24-01303-s001.zip › SupplementarySU_rev.pdf]

Supplementary Information

PEG-OligoRNA hybridization of mRNA for developing sterically stable lipid nanoparticles toward *in vivo* administration

Shota Kurimoto, Naoto Yoshinaga, Kazunori Igarashi, Yu Matsumoto, Horacio Cabral and Satoshi Uchida

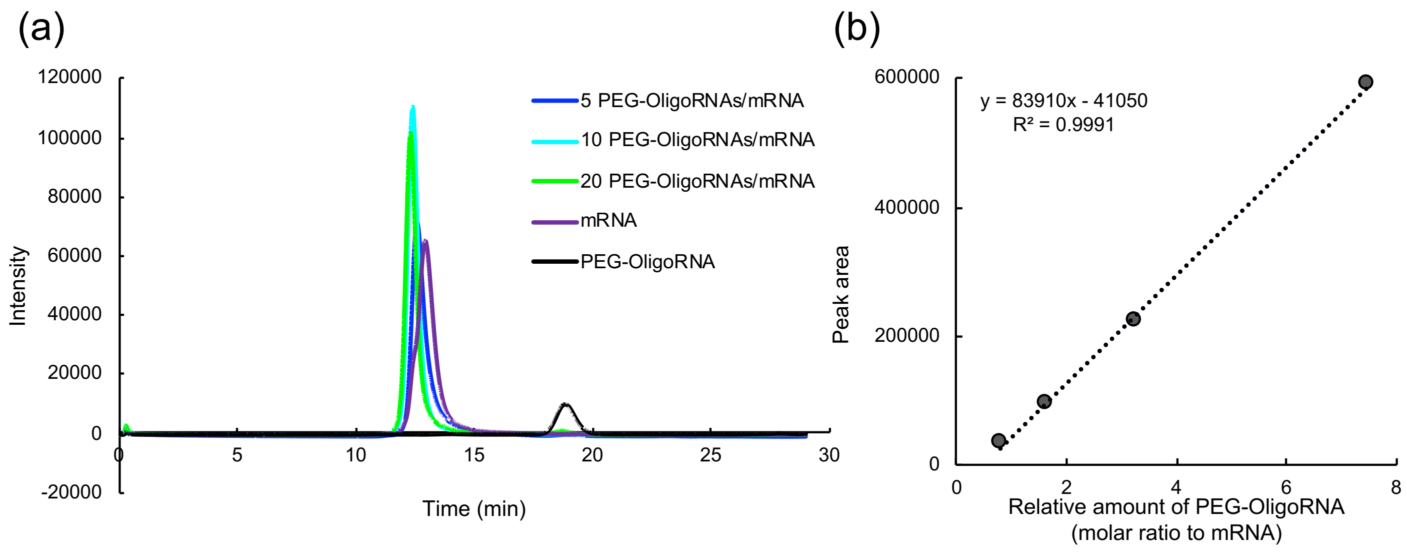

Figure S1. Gel permeation chromatography (GPC) for evaluation of hybridization efficiency. GPC was performed using JASCO UV-4075 (JASCO, Tokyo, Japan) equipped with Sephadex 200 Increase 10/300 GL column (GE Healthcare UK Ltd., Buckinghamshire, UK) in 10 mM PBS at flow rate of 0.75 ml/min. (a) GPC chart of mRNA hybridized with 5, 10 or 20 PEG-OligoRNAs, free mRNA and free PEG-OligoRNA. The peaks observed in 12 – 15 min are derived from mRNA or PEG-OligoRNAs/mRNA, and those in 18 – 20 min are from free PEG-OligoRNA. (b) Peak area from PEG-OligoRNA with different concentration. The amount of free PEG-OligoRNA in the solution of PEG-OligoRNAs/mRNA was calculated based on peak area from free PEG-OligoRNA in (a) and standard curve prepared in (b).

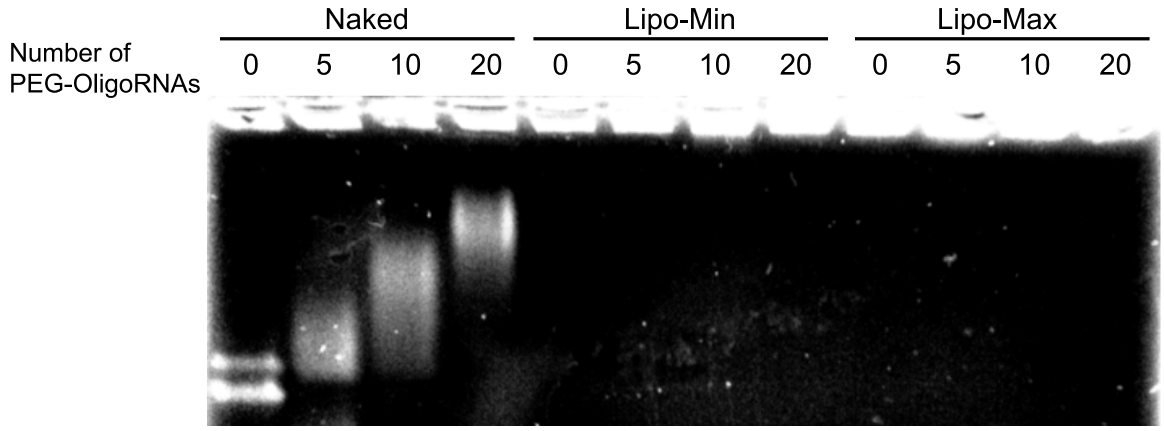

Figure S2. Gel electrophoresis of lipofectamine LTX/mRNA LNPs. Two LNP formulations were prepared by mixing mRNA with minimal or maximal volume of lipofectamine LTX solution that the manufactures suggest to use (Lipo-Min and Lipo-Max, respectively). Unhybridized mRNA and mRNA hybridized with 5, 10, or 20 PEG-OligoRNAs were subjected to electrophoresis, in a naked form or in a form of Lipo-Min or Lipo-Max.

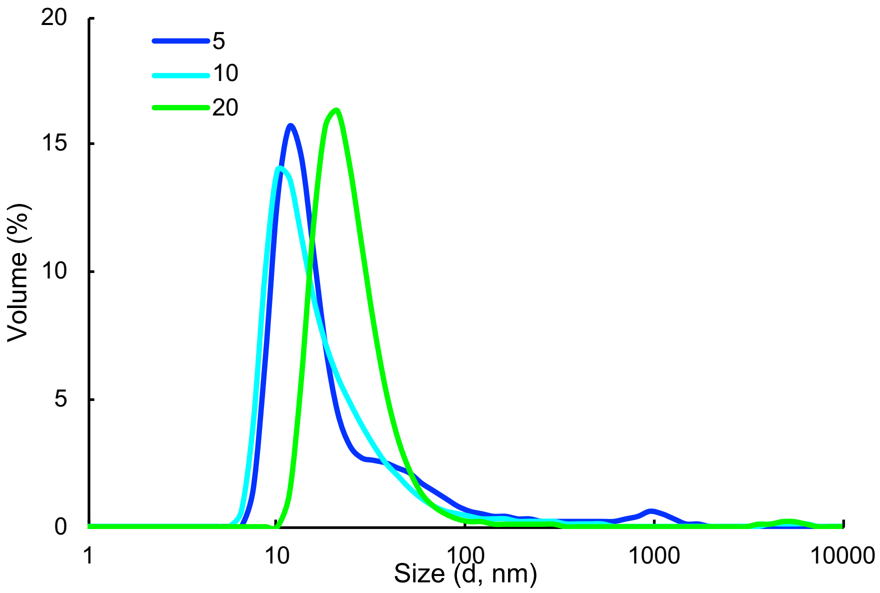

Figure S3. Dynamic light scattering (DLS) measurement of Lipofectamine LTX added with PEG-OligoA. PEG-OligoA was added to Lipofectamine LTX without the presence of mRNA. The concentration of PEG-OligoA and Lipofectamine LTX in this experiment was adjusted to the same as that of PEG-OligoRNAs and Lipofectamine LTX used to obtain **Figure 4d**. The numbers, 5, 10, and 20 in this figure, represent eq. of PEG-OligoA relative to mRNA concentration in the experiment of **Figure 4d**, respectively.

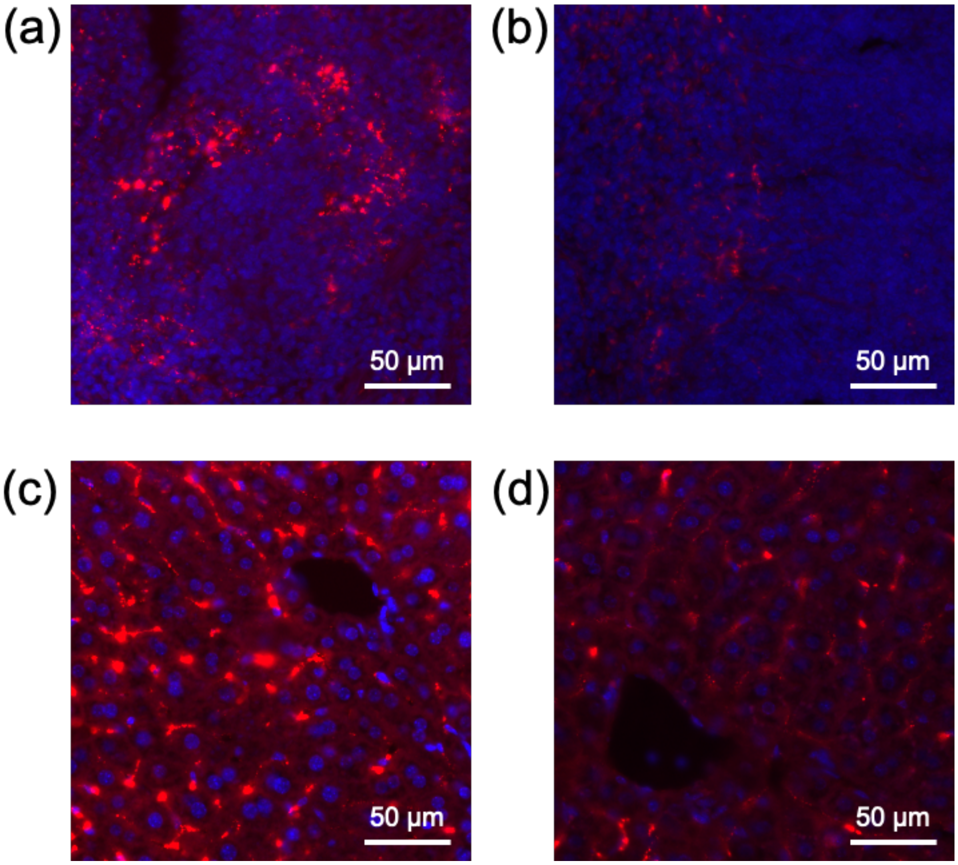

Figure S4. Distribution of Lipo-Max in the spleen and the liver. Lipo-Max prepared from Cy5-labeled mRNA (red) was intravenously injected to the mice. Five min after the injection, (**a**, **b**) the spleen and (**c**, **d**) the liver was excised for preparation of frozen tissue sections. Blue: nucleus (DAPI). (**a**, **c**) Lipo-Max loaded with non-PEGylated mRNA. (**b**, **d**) Lipo-Max loaded with 20 PEG-OligoRNAs/mRNA.

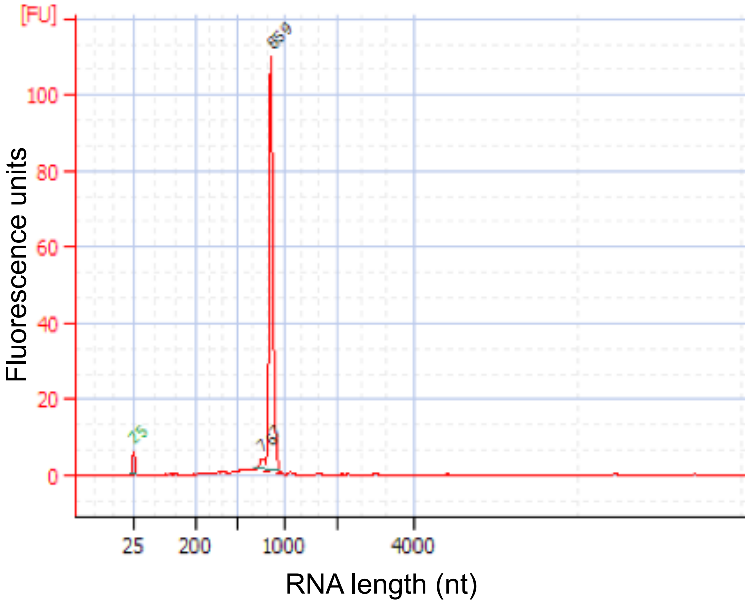

Figure S5. Capillary electrophoresis of *GLuc* mRNA. *GLuc* mRNA was subjected to the electrophoresis after mixed with 25 nt RNA marker.

1 GGGAGACCGGCCUCGAGCAGCUGAAGCUUGGUACCGAGCUCGGAUCCAGCCACCAUGG**GAGUCAAAGUUCUGUUU**GCCCUGAUCU**GCAUCGCUGUGGCCG**

1 ((....)).[[...((((.))....))]]..[[[(((((((((([[((..(((((.(((..................((((((((...........(((.[

101 **A**GGCCAAGCCCACCGAG**AACAACGAAGACUUCAA**CAUCGUGGCCGUGG**CCAGCAACUUCGCGACC**ACGGAUCUCGAUGCUG**ACCGCGGGAAGUUGCCC**GG

101 [[]]][[..[[...[[[............((((..(((((...((((((...(([[....]]..))))))....))))).........))))))....((

201 CAAGAAGCUGCCG**CUGGAGGUGCUCAAAGA**GAUGGAAGCCAAUG**CCCGGAAAGCUGGCUGC**ACCAGGGGCUGUC**UGAUCUGCCUGUCCCAC**AUCAAGUGC

201 (........)))..[[[.[[[.[[[[...[[.[[[......((.....))..

301 ACGCCCA**AGAUGAAGAAGUUCAUC**CCAGGACGCUGCCACACCUA**CGAAGGCGACAAAGAGU**CCGCACAGGGCGGCAUAGGCGAGGCGAUC**GUCGACAUUC**

301 ........((((((....)))))).......[[[[[[.............................((.))]]]]...((((()))))]]]]]]]]]]]]

401 **CUGAGAU**UCCUGGGUUCAAGGACU**UGGAGCCCAUGGAGCAG**UUCAUCGC**ACAGGUCGAUCUGUGUG**UGGACUGC**ACAACUGGCUGCCUCAA**AGGGCUUG**C**

401 [[[[[..[[[((((((...))))))]]]]]]]]]]].((((((((.((((((((....))))))))))))))))......(((.(()))...))]]]]]]

501 **CAACGUGCAGUGUUCU**GACCUGCU**CAAGAAGUGGCUGCCGC**AACGCUGUG**CGACCUUUGCCAGCAAG**AUCCAGGG**CCAGGUGGACAAGAUCA**AGGGGGCC

501)..[[[[[[((((..)).........................))]]]]]]]]..]]].........))).)))))))))))))).....]]......((((

601 GGUGGUGACUAAGCGGCCGCU**CGAGCAUGCAUCUAGAG**GAUCCCCGG**GUACCGAGCUCGAAUUC**AAAAAAAAAAAAAAAAAAAAAAAAAAAAAAAAAAAA

601 (............)))))..........................))]]])))))))))))...

701 AAAAAAAAAAAAAAAAAAAAAAAAAAAAAAAAAAAAAAAAAAAAAAAAAAAAAAAAAAAAAAAAAAAAAAAAAAAAAAAAAAA

701 ....

Table S1. Secondary structure in *GLuc* mRNA sequence. The structure was predicted using IPKnot software (http://rtips.dna.bio.keio.ac.jp/ipknot/). "( )" , "[ ]" showed base paring and ".." showed the bases without base pairing. Bold characters represent the sequence to which complementary PEG-OligoRNAs were designed.
